# Effects of Oligosaccharides Isolated From Pinewood Hot Water Pre-hydrolyzates on Recombinant Cellulases

**DOI:** 10.3389/fbioe.2018.00055

**Published:** 2018-05-15

**Authors:** Hong Fang, Gurshagan Kandhola, Kalavathy Rajan, Angele Djioleu, Danielle Julie Carrier, Kendall R. Hood, Elizabeth E. Hood

**Affiliations:** ^1^Department of Biology, Molecular Biosciences Graduate Program, College of Biology, Molecular Biosciences, Arkansas State University, Jonesboro, AR, United States; ^2^Department of Biological & Agricultural Engineering, University of Arkansas, Fayetteville, AR, United States; ^3^Department of Biosystems Engineering and Soil Science, University of Tennessee, Knoxville, TN, United States; ^4^Infinite Enzymes, Arkansas State University, Jonesboro, AR, United States; ^5^Arkansas State University Biosciences Institute and College of Agriculture and Technology, Arkansas State University, Jonesboro, AR, United States

**Keywords:** loblolly pinewood, hot water pretreatment, centrifugal partition chromatography, recombinant cellulase, cellulolytic enzymes inhibition, cellulose digestion

## Abstract

Loblolly pine residues have enormous potential to be the raw material for advanced biofuel production due to extensive sources and high cellulose content. Hot water (HW) pretreatment, while being a relatively economical and clean technology for the deconstruction of lignocellulosic biomass, could also inhibit the ensuing enzymatic hydrolysis process because of the production of inhibitors. In this study, we investigated the effect of oligosaccharide fractions purified from HW pre-hydrolyzate of pinewood using centrifugal partition chromatography (CPC) on three recombinant cellulolytic enzymes (E1, CBHI and CBHII), which were expressed in the transgenic corn grain system. The efficiency of recombinant enzymes was measured using either a 4-methylumbelliferyl-β-D-cellobioside (MUC) or a cellulose-dinitrosalicylic acid (DNS) assay system. The results showed that HW pre-hydrolyzate CPC fractions contain phenolics, furans, and monomeric and oligomeric sugars. Among CPC fractions, oligomers composed of xylan, galactan, and mannan were inhibitory to the three recombinant enzymes and to the commercial cellulase cocktail, reducing the enzymatic efficiency to as low as 10%.

## Introduction

Considering the large volume of logging residues of both softwood and hardwood from existing wood processing plants, the forestry industry has recently expressed strong interest in becoming providers of biomass for bioenergy (heat and power generation) and biofuel production (Bioenergy, 2008). Loblolly pine (*Pinus taeda* L.) in particular, is the principal commercial softwood of the Southeastern United States and was estimated to generate between 55 and 93 metric tons of logging residues in 2009 (Eisenbies et al., 2009). Therefore, loblolly pine logging residues could be utilized as raw material for advanced biofuel production. To overcome the natural recalcitrance of lignocellulosic biomass and make it more accessible to the cellulolytic enzymes, different pretreatments including biological, chemical, physical, and thermal processes have been applied to the raw materials prior to biochemical saccharification (Yang and Wyman, 2008; Agbor et al., 2011). Unfortunately, during the pretreatment process, a number of byproducts are formed from the degradation of holocellulose and lignin such as phenolics, furans, organic acids, and unfermentable monomeric and oligomeric sugars (xylose, mannose, xylan, mannan, etc.). All these compounds have been shown to negatively affect the saccharification step during biofuel production by inhibiting the cellulolytic enzymes (Duarte et al., 2012; Gao et al., 2014; González-Bautista et al., 2017).

Hot water (HW) treatment is one of the pretreatment methods that allows the use of water (at temperatures above 150°C and various pressures) as a solvent and reaction medium for biomass conversion (Kruse and Dinjus, 2007). Compared to other pretreatment methods or reagents, HW is relatively economical and environmentally-friendly as it does not introduce deleterious chemicals to the liquid processing stream (Yang and Wyman, 2008). HW pretreatment of woody biomass including pinewood has been reported to result in substantial hemicellulose depolymerization and degradation, thereby generating numerous byproducts, such as oligosaccharides, organic acids and furans (Xiao et al., 2011; Yan and Liu, 2015; Rajan and Carrier, 2016; Kandhola et al., 2017). These natural inhibitory byproducts have been reported to reduce cellulolytic enzyme activity (Arora et al., 2012; Duarte et al., 2012; Qi et al., 2014). Specifically, xylose oligomers resulting from hemicellulose depolymerization, were determined to display more enzymatic hydrolysis inhibition than monosaccharides and xylan (Qing et al., 2010). Unlike herbaceous and hardwood biomass, the major polymer units found in pinewood hemicelluloses are O-acetyl-galactoglucomannan and arabino-4-O-methylglucurono-D-xylans (Jönsson and Martín, 2016) and so far, the effect of these component galactoglucomannan oligosaccharides on cellulase efficiency has not been investigated. Given that oligosaccharides are inhibitory to saccharification enzymes (Qing et al., 2010) and that pine hemicelluloses have different composition, there is a knowledge gap as to the effect of pine-derived oligosaccharides on cellulolytic enzyme functionality.

Cellulolytic enzymes have been isolated from a wide range of microorganisms (Henrissat and Bairoch, 1993). For instance, six bacterial endo-cellulases (E1-E6) have been isolated from *Thermomonospora fusca*, and two fungal exo-cellulases (cellobiohydrolase CBHI and CBHII) have been isolated from *T. fusca* and *Trichoderma reesei* (Irwin et al., 1993). The activity of these cellulolytic enzymes is inhibited by rice straw derived HW pre-hydrolyzates (Rajan and Carrier, 2014). Although bacterial and fungal derived cellulolytic enzymes have been used to examine the inhibitory effect of HW pretreatment generated byproducts, they cannot provide detailed inhibitory information because these enzymes are produced as mixtures instead of pure enzyme. In recent years, a transgenic corn expression system has been successfully used to produce recombinant enzymes like endo-1,4-β-D-glucanase (E1), 1,4-β-D-glucan-cellobiohydrolase I (CBHI), and 1,4-β-D-glucan-cellobiohydrolase II (CBHII) (Hood et al., 2007; Devaiah et al., 2013). These recombinant cellulolytic enzymes are advantageous for investigating cellulase synergism, inhibitor identification and large-scale industrial usage, because they are single activity preparations.

In this study, we investigated the effects of pine-derived oligosaccharides that are present in liquid HW pre-hydrolyzates on model cellulolytic enzyme systems, such that the knowledge gap between single enzyme functionality and pine-derived oligomers could be bridged. Different recombinant cellulolytic enzymes, including *Acidothermus cellulolyticus* derived E1, and *T. reesei* derived CBHI and CBHII were expressed in transgenic corn grain. The purified recombinant enzymes were tested alongside a commercial cellulase cocktail (Sigma C2730) for the digestion of substrates like 4-methylumbelliferyl-β-D-cellobioside (MUC) and commercial standard cellulose (Sigmacell cellulose). In order to acquire more detailed information on cellulase inhibition, the liquid pre-hydrolyzate derived from HW pretreatment of pinewood was fractionated into different components using centrifugal partition chromatography (CPC) and the fractions were tested separately.

## Materials and methods

### Biomass

Loblolly pine (*P. taeda*) was grown in the School of Forestry & Natural Resources POW Camp, in Monticello, AR (33°43′51′′ N, 91°43′50′′ W) and harvested in September 2015. The harvested stem wood had a diameter measured at 1.37 m above the ground of 0.31–0.36 m. The particle size of debarked wood was reduced using a laboratory wood chipper and chips of size 2.0–3.5 mm that passed through and were retained between US 6 and 10-mesh sieves, respectively, were separated and stored at −30°C until further use.

For compositional analysis, the pine chips were air-dried to a moisture content of <10%, ground using a Thomas Wiley® Mini-Mill (Swedesboro, NJ), and passed through a 20-mesh screen to obtain particles of 0.263 mm in size. The ethanol extractives, structural carbohydrates, and lignin content of pine biomass were determined as described in the NREL (National Renewable Energy Laboratory, Golden, CO) laboratory analytical protocols (LAPs) (NREL/TP-510-42618 and 510-42619).

### Chemicals

For the characterization of CPC fractions, commercial standards of glucose, cellobiose, and 5-hydroxymethylfurfural (HMF) were purchased from Alfa-Aesar (Haverhill, MA); xylose, galactose, arabinose, mannose, and furfural were purchased from Sigma-Aldrich (C2730, St. Louis, MO). Standards for acetic acid and formic acid were purchased from Amresco (Solon, OH). Folin-Ciocalteu's (F-C) phenol reagent and sulfuric acid (98%) were obtained from Thermo Fisher Scientific (Fair Lawn, NJ).

Recombinant enzymes were purified from transgenic corn grain. A commercial *T. reesei* cellulase cocktail was purchased from Sigma-Aldrich (C2730, St. Louis, MO). Four-methylumbelliferyl-β-D-cellobioside (MUC) (Gold Biotechnology) and 4-methylumbelliferone (MU) (Sigma-Aldrich, M1381) were used in the MUC assay as the substrate and for standard curve construction. Sigmacell cellulose (Sigma-Aldrich, S3504), 3,5-dinitrosalicylic acid (DNS) (Sigma-Aldrich, 128848) and D-glucose (Fisher Scientific) were used in the cellulose-DNS assay as the substrate and for standard curve construction.

### HW pretreatment of pinewood

The pinewood chips were loaded in a 1 L stirred Parr reactor (Model 4525, Moline, IL) and mixed with water at 17% (w/v) ratio. The reactor was heated up to and held at 180°C for 30 min. At the end of the reaction, the reactor was immediately cooled and the liquid hydrolyzate was recovered using a Buchner filtration apparatus fitted with a Whatman #1 filter paper. The HW pretreatment corresponded to a combined severity factor of 0.58. While the initial pH of the biomass slurry was 7.0, the average pH at the end of HW pretreatment was 3.25. The liquid hydrolyzate was frozen and then lyophilized at −44°C and 7.7 Pa, in a FreeZone 12 L console freeze dry system (Labconco®, Kansas City, MO) for 72 h. The HW pre-hydrolyzate was further characterized using high performance liquid chromatography (HPLC) analysis and used for enzyme inhibition studies.

### Centrifugal partition chromatography (CPC) fractionation

A biphasic solvent system composed of butanol, methanol, and water at a 5:1:4 (v/v/v) ratio was used for CPC fractionation of pinewood pre-hydrolyzate. A Gilson PLC 2050 preparatory HPLC system was connected to a 250 mL CPC rotor (Armen Instrument, Saint-Avé, France) that was operated in ascending mode at 2,300 rpm. Two detectors were used: an analog input at 254 nm to detect UV absorbing compounds and an Evaporative Light Scattering Detector (SofTA Corp, Westminster, CO) to monitor carbohydrates. For each trial, 5 g of HW pre-hydrolyzate was dissolved in 30 mL of the aqueous phase and injected into the rotor and eluted at 8 mL/min. The total run time was 280 min and the collected fractions were dried in a Savant™ SPD1010 concentrator (Thermo Scientific, Ashville, NC) at 0.7 kPa for 8 h. The CPC fractions were characterized and then consolidated according to their composition.

### Characterization of pinewood CPC fractions

The total phenolic content of the CPC fractions was determined using the F-C reagent, where an aliquot of 0.5 g/L was mixed with 0.2 N F-C phenol reagent and incubated in the dark. Color was developed by addition of 7.5% sodium carbonate solution and after 2 h incubation in the dark. The sample absorbance at 765 nm was measured using a spectrophotometer (Model 517601, Beckman Coulter Inc., Indianapolis, IN) and expressed in gallic acid equivalent.

CPC fractions rich in carbohydrates, furans and organic acid were analyzed using HPLC. Monomeric sugars (xylose, glucose, galactose, mannose, arabinose) and oligomeric sugars (galactan, glucan, mannan) were analyzed on a Waters Alliance 2695 system (Milford, MA) equipped with an SP0810 (Shodex, Kawasaki, Japan) analytical column and a refractive index detector (Waters 2414, Milford, MA). The oligomeric sugars were digested in 72% sulfuric acid and converted to monomeric sugars prior to HPLC analysis according to NREL/TP-510-42618. Furans and organic acids were analyzed using a Bio-Rad Aminex HPX-87H analytical column and monitored using a photodiode array detector (Waters 2996, Milford, MA) set at wavelengths of 210 nm. Methods for HPLC analysis of sugars, furans and organic acids were based on the NREL/TP-510-42623.

### Recombinant cellulase preparation

Three recombinant cellulolytic enzymes including E1 from *A. cellulolyticus*, and CBHI and CBHII from *T. reesei* were expressed in transgenic corn. Specifically, E1 and CBHI were expressed in the seed germ while CBHII was expressed in the endosperm. Two kilograms of transgenic corn grain (CBHII) or corn germ (E1 and CBHI) were ground with a coffee grinder (Cuisinart) in small batches (~100 g each) for about 30 s and the entire batch was mixed with 5 L 0.05 M sodium acetate extraction buffer, pH 5.0, for 2 h using an electric mixer at 4°C. The corn slurry was mixed with 1.2 kg of Hyflo® Super Cel® (Celite Corporation, Lompoc, CA) and filtered through a 24-cm diameter Buchner funnel with a paper filter to make the crude extract.

CBHI was purified from its crude extract exactly according to the steps described by Hood et al. (2014). For E1, the crude extract was heated to 60°C for 1 h to allow the precipitation of native corn protein. After heating, the precipitate was removed via filtration as above using a 15 cm Buchner funnel with a glass fiber filter. The filtrate underwent a salting out step by adding 70% ammonium sulfate (AS) and stirring on ice for 1 h. The precipitate was rinsed with additional extraction buffer (400–500 mL) containing 70% AS. After rinsing, the precipitate was dissolved in 50 mM sodium acetate buffer, pH 4.0, filtered through a Buchner funnel, and desalted using tangential flow filtration (TFF, Millipore, Billerica, MA). Diafiltration was accomplished using a 10 kDa MWCO (molecular weight cut off) Biomax membrane (polyethersulfone) and two volumes of 50 mM sodium acetate buffer, pH 4.0. The subsequent column purification was the same as described by Hood et al. (2014). For CBHII, the crude extract was first 2-fold concentrated using tangential flow filtration (TFF, Millipore, Billerica, MA) and then salted out with 70% AS on ice for 1 h. After salting out, the precipitate was rinsed with extraction buffer (400–500 mL) containing 70% AS as above. After subsequent work-up, the diafiltered CBHII extract was then loaded onto a column (1.5 × 30 cm) containing 50 mL Gigacap S650 resin (TOSOH Bioscience, King of Prussia, PA) for purification. All purified enzymes were made into 1 mL stocks containing 20% glycerol and stored at −20°C.

### Recombinant enzyme characterization

Purified recombinant enzymes were characterized by SDS-PAGE-electrophoresis (Schägger and Von Jagow, 1987) and Coomassie Blue staining (Blakesley and Boezi, 1977). A total soluble protein (TSP) assay of each enzyme was done using the Synergy HT microspot reader (BioTek, Winooski VT). The approximated specific activity of purified enzymes was determined by MUC assay (Chou et al., 2011) according to the following formula:

(1) $$\begin{array}{r} Specific\;activity\;\left(U/mg\right)=\frac{P}{tVc} \end{array}$$

where

*U* = activity unit, defined as the amount of enzyme that can hydrolyze 1 nmol of MUC per minute at 50°C, pH 5.0

*P* = amount of MU released in MUC reaction, nmol

*t* = reaction time, min

*V* = volume of purified enzyme applied in MUC assay, mL

*c* = the TSP concentration of purified enzyme solution, mg/mL.

### Cellulolytic enzyme assays

The MUC and cellulose-DNS assays were utilized to evaluate the inhibition effect of the whole liquid pre-hydrolyzate from the HW pretreatment of pinewood and its CPC fractions. The enzymatic efficiency (η_e_) in either the MUC assay or cellulose-DNS assay was represented by:

(2) $$\begin{array}{r} \%\;\eta_{e}\;=\;\frac{amount\;of\;product\;molecules\;released\;in\;the\;presence\;of\;inhibitor}{amount\;of\;product\;molecules\;released\;in\;control}\times 100 \end{array}$$

#### MUC (4-methylumbelliferyl-β-D-cellobioside) assay

In the MUC assay, the enzyme activity was determined by quantifying the amount of released MU (Chou et al., 2011). The substrate stock (1 mM) was prepared by first dissolving 25 mg MUC in 5 mL dimethyl sulfoxide and then by adding 45 mL of 0.05 M sodium acetate buffer, pH 5.0. A reaction mixture (Table 1) with a total volume of 120 μL containing 80 μL of MUC stock (3.33 mg/mL of final concentration) and 0.0165 μg of E1, 0.32 μg of CBHI or 1.15 μg of CBHII was prepared in 0.05 M sodium acetate buffer, pH 5.0 and incubated in a 96-well plate, at 50°C for 1 h. At the end of incubation, an 80 μL aliquot of 0.2 M sodium carbonate was added to stop the reaction and the final fluorescent product, MU, was measured using a Synergy HT microplate reader (BioTek) at excitation and emission wavelengths of 350 and 420 nm, respectively. The effect of CPC fractions on MUC activity was determined by testing different concentrations of CPC fractions (1, 2, 3, and 4 mg/mL) in the 120 μL enzymatic reaction system and compared to a control without any CPC fraction. In order to quantify the released MU, a series of MU dilutions with gradient concentrations (0, 0.02, 0.04, 0.06, 0.08, and 0.10 mM) was made for a standard curve.

**Table 1:** Composition of MUC assay with a total volume of 120 μL.

**Enzyme addition**	**Fraction addition**			
	**F1-F6 (1 mg/mL)**	**F1-F6 (2 mg/mL)**	**F1-F6 (3 mg/mL)**	**F1-F6 (4 mg/mL)**
	**mg of fraction per** μ**g of enzyme**			
E1 (0.0165 μg)	7.3	14.6	21.9	29.2
CBHI (0.32 μg)	0.4	0.8	1.2	1.6
CBHII (1.15 μg)	0.1	0.2	0.3	0.4

#### Cellulose-DNS assay

In the cellulose-DNS assay, the enzyme efficiency was determined by quantifying the amount of produced reducing sugar (Hood et al., 2014). For testing CPC fractions, a reaction mixture (Table 2) with a total volume of 200 μL containing 1.0 mg of Sigmacell cellulose and 16.5 μg of E1, 16.0 μg of CBHI, 11.5 μg of CBHII, or 60.0 μg of commercial cellulase cocktail was prepared with 0.05 M sodium acetate buffer, pH 5.0 and incubated in a 96-well plate with 1 glass bead at 50°C for 1 or 2 days (1 day for commercial cellulase cocktail, whereas 2 days for recombinant cellulolytic enzymes) with 100 rpm reciprocal shaking. About 1.0 and 2.25 mg/mL of whole liquid HW pre-hydrolyzate and 1.5 mg/mL of each CPC fraction were tested in this assay respectively, being controlled by reactions without any inhibitors. After reaction completion, 50 μL of reaction solution was collected from each sample well in order to determine the amount of reducing sugar produced. In order to quantify the produced reducing sugar, a series of glucose solutions with gradient concentrations (0, 0.4, 0.8, 1.6, 2.4, 3.2, 4.0, and 8.0 mg/mL) were made for the standard curve.

**Table 2:** Composition of cellulose-DNS assay with a total volume of 200 μL.

**Enzyme addition**	**Fraction addition**		
	**F1-F6 (1.5 mg/mL)**	**Whole HW pre-hydrolyzate (1.0 mg/mL)**	**Whole HW pre-hydrolyzate (2.25 mg/mL)**
	**mg of fraction per** μ**g of enzyme**		
E1 (16.5 μg)	0.0182	0.0121	n/a
CBHI (16.0 μg)	0.0187	0.0125	n/a
CBHII (11.5 μg)	0.0261	0.0174	n/a
Commercial cellulase cocktail (60.0 μg)	0.0050	0.0033	0.0075

Because the CPC fractions and whole liquid HW pre-hydrolyzate contain polysaccharide and reducing compounds that would be detected by the DNS assay (Miller, 1959), an additional cellulose-free control (substrate-free control) group was set up to account for the background absorbance.

### Statistical analysis

For the cellulolytic enzyme assay, experiments were performed with at least three replicates for MUC assay and at least five replicates for cellulose-DNS assay, and data were averaged with experimental standard error. A double tailed *t*-Test was used for statistical significance, *p* < 0.05.

## Results

### Cellulolytic enzyme purification

**Figure 1:**
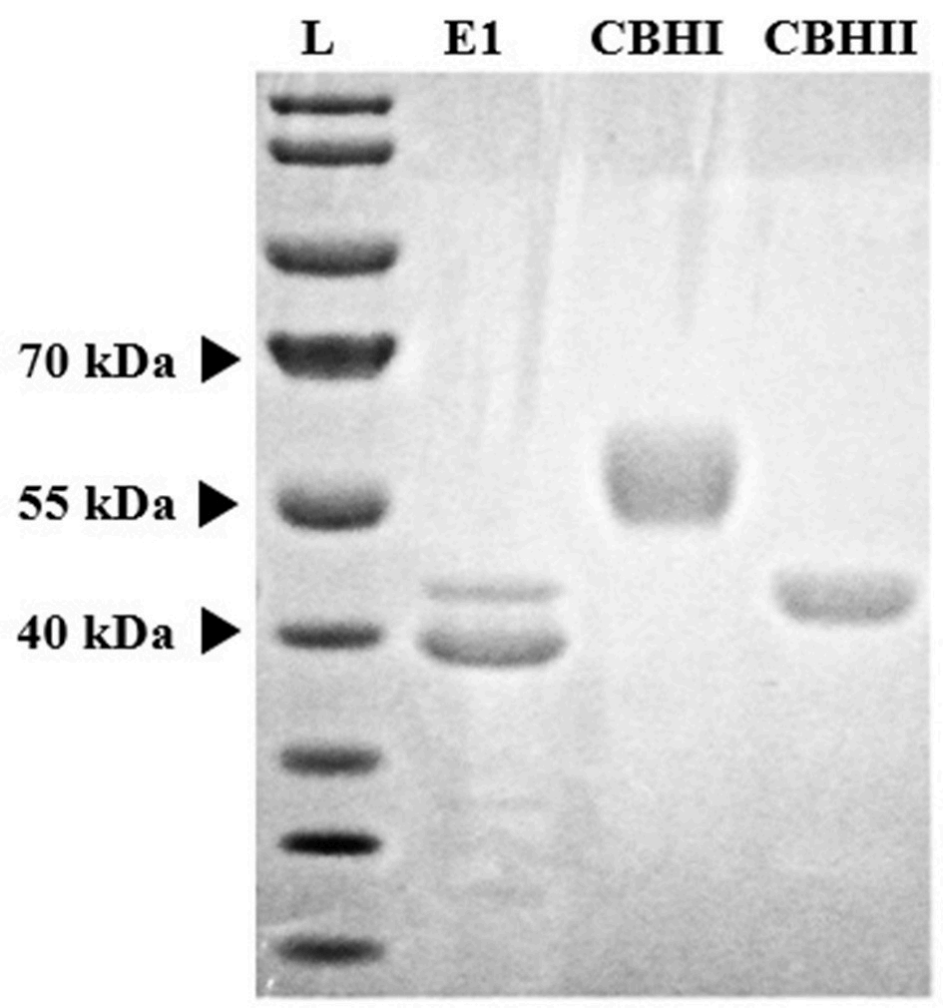
Coomassie Blue stained SDS-PAGE gel of three recombinant cellulolytic enzymes purified from corn grain expression system. Lane L is the standard protein ladder.

Recombinant cellulolytic enzymes i.e., E1, CBHI, and CBHII were expressed in and extracted and purified from the transgenic corn grain system. The approximate molecular size of each purified enzyme (E1, ~40 kDa; CBHI, ~53 kDa; and CBHII, ~42 kDa), was determined by SDS-PAGE electrophoresis (Figure 1) and corresponded with the sizes reported previously (Hood et al., 2007; Devaiah et al., 2013). Among them, E1 presented two bands (a lower dark band and an upper pale band) around 40 kDa. Even though a cleavage is implied in the E1 protein, purified E1 retains considerable activity. Purification yield was expressed as the ratio of TSP content of each purified enzyme to the total mass of corn grain. Compared with E1 or CBHI, CBHII showed a less intense band indicating its relatively lower purification yield, which was about half of E1 and CBHI and was confirmed by the TSP assay (Table 3). The purified recombinant cellulolytic enzymes were tested using the MUC assay to determine their baseline activities. The specific activity (one unit is defined as the amount of enzyme that can hydrolyze 1 nmol of MUC per minute at 50°C, pH 5.0) of each purified enzyme was determined and shown in Table 3.

**Table 3:** Characterization of cellulolytic enzymes expressed and purified from transgenic corn.

**Enzyme**	**Molecular weight (kDa)**	**Purification yield (mg enzyme/g corn grain)**	Specific activity (U/mg)^*^
E1	40	0.48	1436
CBHI	53	0.48	90
CBHII	42	0.28	35

* *1U, the amount of enzyme that can hydrolyze 1 nmol of MUC per minute at 50°C, pH 5.0*.

### Preparation of pinewood liquid HW pre-hydrolyzates

The raw pine chips were determined to be composed of 38% glucan, 36% lignin, 11% mannan, 7% xylan, 5%, galactan, 1.5%, arabinan, 5% ethanol extractives, and 0.1% ash, on an oven dry basis. Hot water was applied to pretreat the harvested loblolly pinewood. The pretreatment condition was selected based on previous reports (Garrote et al., 1999; Lee et al., 2010; Zhu et al., 2010; Leppänen et al., 2011), because it resulted in sufficiently high hemicellulose yield without extensive degradation of the extracted oligosaccharides into furfural and HMF. HW pretreatment resulted in about 22% mass loss (dry weight) and in removing 3% of glucan and 33% of pine hemicellulose i.e., 37% mannan, 25% xylan, 18% arabinan, and 16% galactan. The liquid hydrolyzate was frozen and then lyophilized after HW pretreatment. Composition of the lyophilized HW pre-hydrolyzate is given in Table 4, which showed that it was composed of 32% monomeric sugars, 35% oligomeric sugars, 15% of formic and acetic acids, and 3% furans. The liquid HW pre-hydrolyzate had a very low total phenolic content of 0.5%.

**Table 4:** Composition of lyophilized pinewood liquid HW pre-hydrolyzate.

**Compound**	**% Dry wt. with standard deviation**	**CPC fraction composition (% total dry wt.)**					
		**F1**	**F2**	**F3**	**F4**	**F5**	**F6**
Xylose	12.0 ± 2.0	–	–	–	37	–	–
Glucose	8.4 ± 4.1	–	–	–	16	–	–
Mannose	6.5 ± 2.2	–	–	3	15	–	–
Galactose	2.8 ± 0.7	–	1	5	13	–	–
Arabinose	2.1 ± 0.3	–	–	–	–	–	–
**OLIGOSACCHARIDES**							
Mannan	19.2 ± 0.8	–	–	–	–	13	13
Cellobiose	8.8 ± 0.7	–	–	–	5	–	–
Galactan	3.2 ± 1.3	–	–	–	–	37	35
Glucan	2.4 ± 0.1	–	–	–	–	13	14
Xylan	1.1 ± 0.0	–	–	–	–	7	40
**ORGANIC ACIDS**							
Formic acid	8.9 ± 1.1	–	25	39	–	–	–
Acetic acid	6.4 ± 0.1	–	9	35	–	–	–
**FURANS**							
Furfural	1.5 ± 0.0	–	–	–	–	–	–
HMF	1.4 ± 0.5	–	33	5	–	–	–
Total phenolics^*^	0.5 ± 0.0	42	2	–	–	–	–
Sum	85.2 ± 5.5						

* *Gallic acid equivalent*.

The use of lyophilized liquid HW pre-hydrolyzates enabled concentration of potential inhibitors prior to CPC fractionation. Analysis showed that the lyophilization process did not significantly affect the composition of the pine HW pre-hydrolyzates. The yield of lyophilized HW pre-hydrolyzate was 14.5% (w/w) of the original pine biomass, similar to previously reported values of 14–16% for Douglas fir and Eucalyptus (Lee et al., 2010). The different components of liquid HW pre-hydrolyzate were eluted in the following order: phenolics, furans, organic acids, monosaccharides and finally, oligosaccharides. The detailed composition of each CPC fraction is given in Table 4. CPC fraction F1 was enriched in phenolic compounds, F2 contained a mixture of furans, organic acids, and some phenolics, F3 was enriched in organic acids and fractions F4, F5, and F6 were enriched in sugars. CPC fraction F6 was particularly enriched in oligomers of xylan (Table 4).

### Effect of CPC fractions on cellulolytic enzyme efficiency

**Figure 2:**
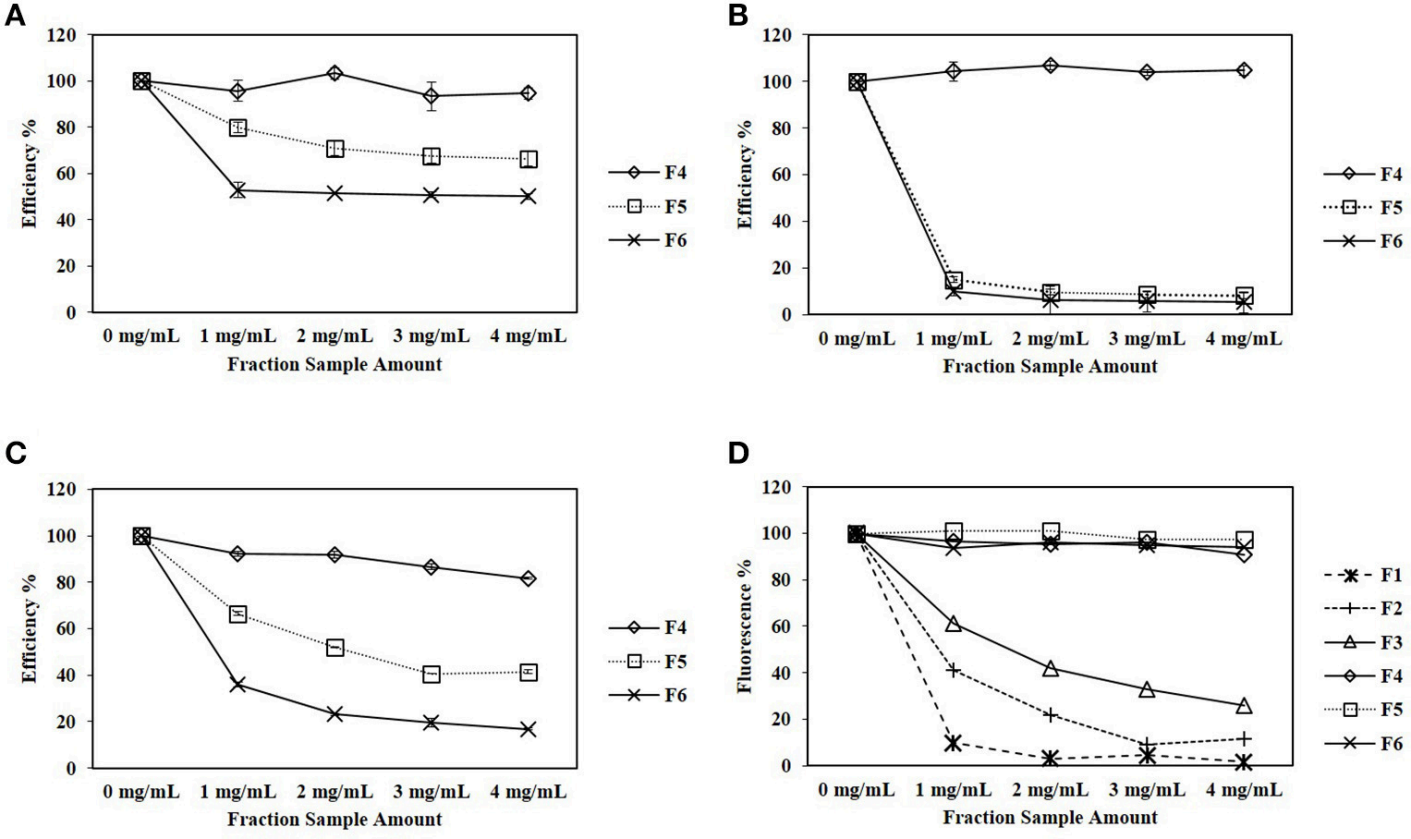
**(A–C)** Effects of CPC fractionated pinewood HW pre-hydrolyzates on E1, CBHI and CBHII enzymatic efficiency during the MUC assay. **(D)** Effects of CPC fractions directly on MU fluorescence without enzyme. Standard error bars in **(A–C)** were used to represent the deviation.

In the MUC assay, each CPC fraction at different concentrations ranging from 0 to 4 mg/mL was tested (Table 1). The assays were set up so that the amount of MUC hydrolyzed was within the standard curve of the assay, and thus the amount of inhibitor per microgram of each enzyme varied. The enzymatic efficiency of E1, CBHI, and CBHII is presented in Figure 2. The oligomeric sugar-rich fractions, F5 and F6, exhibited a significant inhibitory effect against E1, CBHI, and CBHII. For E1 (Figure 2A), 2 mg/mL of fraction F5 reduced the efficiency to 71%; 1 mg/mL of xylan-rich fraction F6 reduced the efficiency to 53%; whereas the monomeric sugar-rich fraction (F4) did not significantly affect the enzymatic efficiency. For CBHI (Figure 2B), 1 mg/mL of both F5 and F6 fractions were severely inhibitory to the enzymatic efficiency, reducing efficiency to 10%. For CBHII (Figure 2C), the monomeric sugar-rich fraction (F4) slightly reduced the enzymatic efficiency to 82% at 4 mg/mL, whereas the F5 fraction reduced the efficiency to 41% at 3 mg/mL and the F6 fraction reduced the efficiency to 23% at 2 mg/mL. In summary, comparing the sugar-rich fractions, the xylan-rich fraction F6 affected the efficiency of E1, CBHI and CBHII the most, followed by fraction F5, while fraction F4 showed mild negative effects only on CBHII. In this test, the effects of phenolics, furans, and organic acid-rich fractions on the cellulolytic enzymes remain unclear because these fractions strongly influenced the fluorescence reading of MU (Figure 2D).

**Figure 3:**
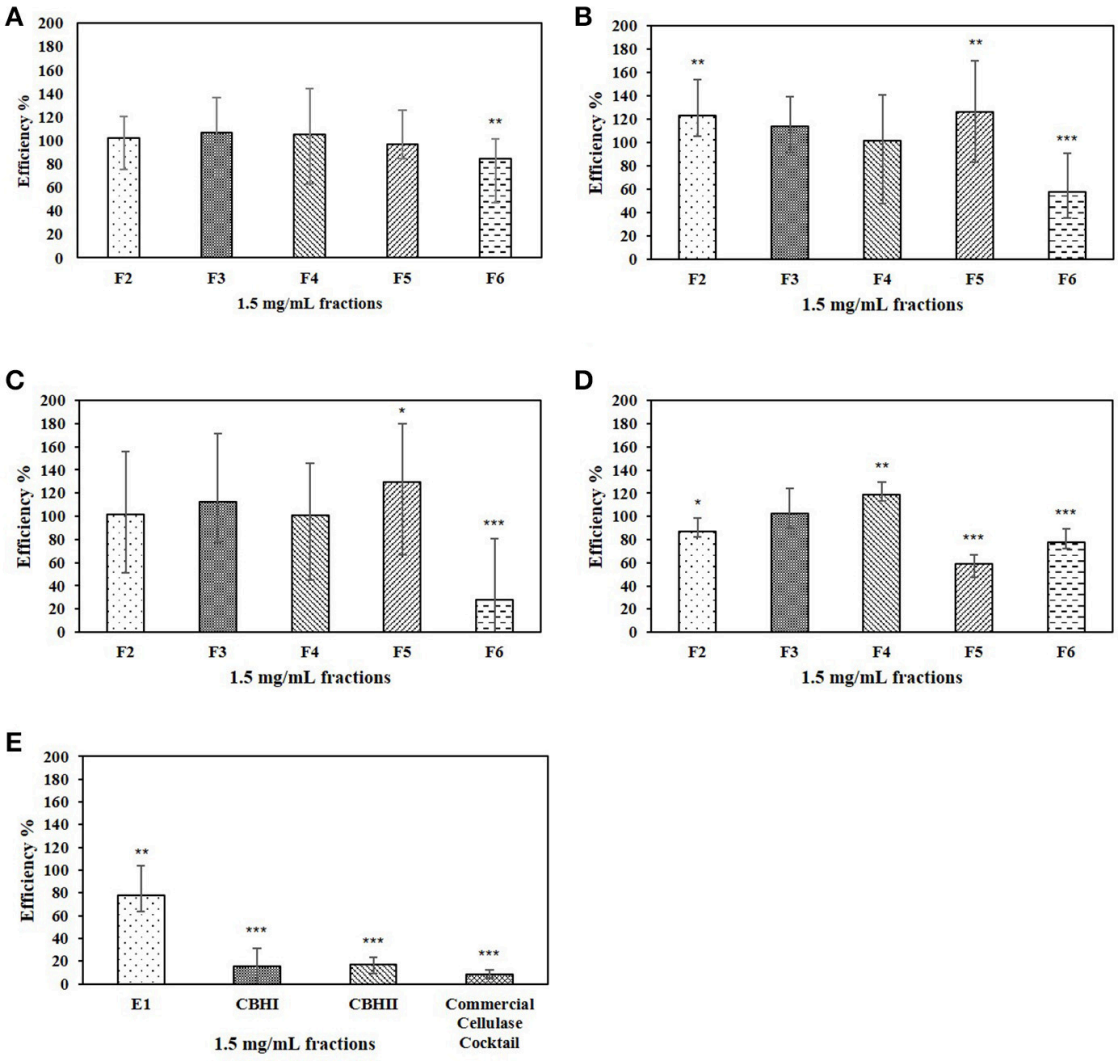
**(A–D)** Effects of CPC fractionated pinewood HW pre-hydrolyzate with a final concentration of 1.5 mg/mL on the enzymatic efficiency of E1, CBHI, CBHII and commercial cellulase cocktail during the cellulose-DNS assay; **(E)** Effect of 10% butanol solvent on the enzymatic efficiency of each cellulolytic enzyme. Symbol * represents the level of significance according to the double tailed *t*-test. Standard error bars were used to represent the deviation.

In the cellulose-DNS assay, 1.5 mg/mL of each CPC fraction was tested against E1, CBHI, CBHII, and commercial cellulase cocktail (Table 2). The enzymatic efficiency of each enzyme is shown in Figure 3. For E1 (Figure 3A), fractions F2, F3, F4, and F5 did not significantly affect enzymatic efficiency, while fraction F6 significantly decreased the enzymatic efficiency to 85%. For CBHI (Figure 3B), fractions F3 and F4 did not significantly affect enzymatic efficiency; fraction F6 significantly decreased the efficiency to 58%. Interestingly, furans and oligomeric sugar-rich fractions (F2 and F5) significantly increased the efficiency of CBHI to 123 and 126%, respectively. For CBHII (Figure 3C), fractions F2, F3, and F4 did not significantly affect the enzymatic efficiency; fraction F6, rich in xylan oligomers, significantly decreased the enzymatic efficiency to 28%. Similarly to CBHI, the small oligomeric sugar-rich fraction (F5) significantly increased the enzymatic efficiency to 130%. For commercial cellulase (Figure 3D), the organic acid-rich fraction (F3) did not significantly affect the enzymatic efficiency, whereas fractions F2, F5, and F6 significantly decreased the enzymatic efficiency to 87, 59, and 77%, respectively. The monomeric sugar-rich fraction (F4) significantly increased the commercial cellulase enzymatic efficiency to 119%.

### Effect of whole liquid HW pre-hydrolyzate on cellulolytic enzyme efficiency

**Figure 4:**
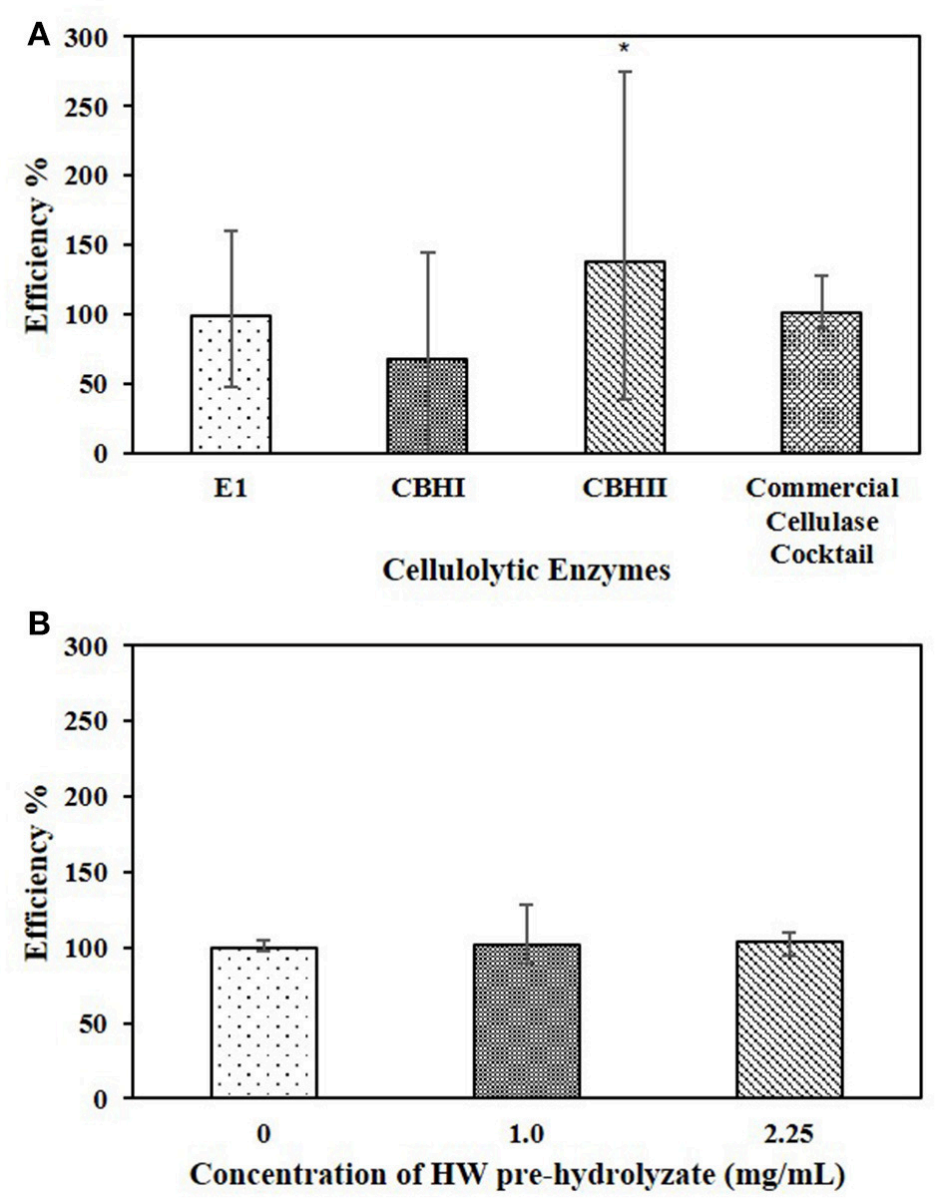
**(A)** Effects of 1.0 mg/mL whole HW pre-hydrolyzate on E1, CBHI, CBHII, and commercial cellulase cocktail during the cellulose-DNS assay. **(B)** Effects of different concentration of whole HW pre-hydrolyzate on commercial cellulase cocktail during the cellulose-DNS assay. Symbol * represents the level of significance according to the double tailed *t*-test. Standard error bars were used to represent the deviation.

The whole liquid HW pre-hydrolyzate, with the concentration of 1.0 mg/mL (Table 2), was tested in the cellulose-DNS assay. The effects of whole liquid HW pre-hydrolyzate on either recombinant cellulolytic enzymes or commercial cellulase cocktail are presented in Figure 4A. The results indicated that, when reacting with Sigmacell cellulose, whole liquid HW pre-hydrolyzate did not significantly affect the efficiency of commercial cellulase cocktail, E1, or CBHI, but significantly increased the efficiency of CBHII to 140%. When the amount of liquid HW pre-hydrolyzate was increased to 2.25 mg/mL and tested against commercial cellulase cocktail, it still did not cause any significant changes in enzymatic efficiency (Figure 4B). Even though the CPC-purified compounds obtained by fractionating the liquid HW pre-hydrolyzate showed differential effects on the cellulolytic enzymes, the whole liquid HW pre-hydrolyzate itself was not detrimental to the specific activity of cellulases.

## Discussion

HW pretreatment is an autohydrolysis process where the cleavage of hemiacetal linkages between hemicellulose and lignocellulose, under high pressure and temperature, results in the formation of acetic acid that catalyzes the formation and removal of oligosaccharides (Mosier et al., 2005). Autohydrolysis also facilitates the subsequent depolymerization of oligosaccharides to monosaccharides or further degradation to aldehydes and ketones. In this instance, in addition to hemicellulose depolymerization, there was also depolymerization and degradation of acid-soluble lignin, which generated phenolic components in the liquid HW pre-hydrolyzates of pinewood.

The different compounds occurring in liquid HW pre-hydrolyzates can be purified using CPC by virtue of their distinct solvent partition coefficients (Lau et al., 2013; Rajan and Carrier, 2016). Chen et al. (2015) were the first to use CPC to purify xylooligosaccharides, directly from crude HW pre-hydrolyzates of *Miscanthus* × *giganteus*, with a 4:1:4 (v/v/v) butanol-methanol-water solvent system. Later Rajan and Carrier (2016), utilized CPC to fractionate phenolic compounds, organic acids, furans, monomeric sugars and xylooligosaccharides from crude HW pre-hydrolyzates of rice straw, using a slightly modified butanol-methanol-water solvent system. In this study, using the modified 5:1:4 (v/v/v) butanol-methanol-water solvent system, we have fractionated the liquid HW pre-hydrolyzate of pinewood chips and obtained fractions enriched in phenolic compounds, organic acids, monomeric sugars and oligosaccharides. This is also the first time we report the fractionation of mannan, glucan and galactan oligosaccharides from Loblolly pinewood hydrolyzate using the CPC technique. After the CPC separation, the major hemicelluloses found in pinewood being *O*-acetyl-galactoglucomannan and arabino-4-*O*-methylglucurono-d-xylans (Jönsson and Martín, 2016), yielded fractions that were rich in xylan, galactan, and mannan. Separation of higher DP (degree of polymerization) oligosaccharides and other known inhibitors from biomass hydrolyzates using the CPC technique provides an opportunity to investigate their inhibitory effects on select cellulolytic enzymes.

The different CPC purified fractions of pinewood liquid HW pre-hydrolyzate were tested for inhibitory effect against the efficiency of recombinant E1, CBHI, and CBHII enzymes, as well as a commercial cellulase cocktail, using either MUC or cellulose as substrate. The MUC assay is a fluorometric method that is more sensitive toward cellulase activities (Barr and Holewinski, 2002), whereas the cellulose-DNS assay is a universal method that detects reducing sugars produced by any cellulolytic enzyme. Fluorochrome detection was hampered by some of the CPC fractions during the MUC assay and necessitated the use of the cellulose-DNS assay system for a more reliable comparison of recombinant cellulase efficiencies. In this study, recombinant cellulase E1, CBHI, and CBHII present large error bars in the cellulose-DNS assay compared to the commercial cellulase cocktail (Figure 3). Even though purified recombinant cellulases are advantageous for giving detailed inhibitory information on each enzyme, the pure enzyme in turn may restrict its digestion of native cellulose. Since endoglucanase E1 can cut at random sites within a cellulose fiber while CBHI and CBHII can only cut the cellulose fiber at either reducing or non-reducing ends, different microstructure situations including presence of cellulose fiber terminals, folding of cellulose fibers and interactions of cellulose fibers may easily influence the efficiency of these recombinant cellulases and cause the huge error bar in the cellulose-DNS assay. Therefore, we performed more replicates in this assay to minimize this influence and synthesize both MUC data and cellulose-DNS data to obtain the final conclusion.

By combining the results of MUC and cellulose-DNS assays (Table 5) it can be concluded that monomeric sugars did not significantly impact the efficiency of recombinant enzymes, except for CBHII whose specific activity slightly decreased in the MUC assay. On the other hand, higher DP oligomeric sugars found in the CPC fraction F6 significantly reduced the enzymatic efficiency of the three recombinant enzymes in both the MUC assay and the cellulose-DNS assay. Xylooligosaccharides (DP > 5) isolated from HW pre-hydrolyzates of rice straw were also reported to be highly inhibitory to recombinant CBHI (Rajan and Carrier, 2016), suggesting that the higher concentration of xylans present in F6 could have caused CBH inhibition.

**Table 5:** A summary of the effects of HW fractions on cellulase enzymatic efficiency.

**Fraction**	**Effects on Enzymatic Efficiency**						
	**MUC Assay**			**Cellulose-DNS Assay**			
	**E1**	**CBHI**	**CBHII**	**E1**	**CBHI**	**CBHII**	**Commercial cellulase cocktail**
F1 (Phenolics)	×	×	×	×	×	×	×
F2 (Furans)	×	×	×	°	+	°	**–**
F3 (Organic Acids)	×	×	×	°	°	°	°
F4 (Monosaccharides)	°	°	**–**	°	°	°	+
F5 (Oligosaccharides, DP 2–6)	**–**	**–**	**–**	°	+	+	**–**
F6 (Oligosaccharides, DP ≥ 6)	**–**	**–**	**–**	**–**	**–**	**–**	**–**
Whole HW hydrolyzate	n/a	n/a	n/a	°	°	+	°

*×, Effect on enzymatic efficiency remains unclear*.

*°, No significant effect on enzymatic efficiency*.

*+, A significantly positive effect on enzymatic efficiency (increased efficiency)*.

–*, A significantly negative effect on enzymatic efficiency (decreased efficiency)*.

*DP stands for degree of polymerization*.

Lower DP oligomeric sugars (F5) significantly reduced the enzymatic efficiency of the three recombinant enzymes in the MUC assay, but improved the enzymatic efficiency in the cellulose-DNS assay, except for E1, whose enzymatic efficiency was not affected in cellulose-DNS assays. Since lower DP oligomeric sugars are the end products of E1 catalysis, it could be concluded that reduction in E1 efficiency, observed with the MUC assay, was the result of feedback inhibition, whereas for the cellobiohydrolases (CBH1 and CBHII) in the cellulose-DNS assay, the added lower DP oligomeric sugars (F5) might have functioned as a substrate for catalysis and thus seemingly enhanced their efficiency. One possible inference for the enhanced enzymatic efficiency is that since overloading cellulolytic enzymes per unit of cellulose would decrease its efficiency, lower DP oligosaccharides that possess structural similarities with cellulose can compete for the free cellulolytic enzymes and therefore relieve the enzyme overload on cellulose fiber and increase its efficiency (Eriksson et al., 2002; Igarashi et al., 2011). Since MUC, instead of cellulose fiber, is the only substrate in the MUC assay, this apparent increase in enzymatic efficiency was not observed in the case of the MUC assay. Based on the cellulose-DNS assay results, it can possibly be inferred that the recombinant CBHI and CBHII are not necessarily inhibited by low concentrations (1.5 mg/mL) of short-chained (DP 2–5) oligomeric sugars.

Although the focus of this work was on oligosaccharides, phenolics, furans and organic acids derived from pinewood were also tested. In the MUC assay, the effects of CPC fractions F1, F2, and F3, rich in phenolics, furans and organic acids, remained unclear because of the severe interference presented by these fractions during the detection of the fluorochrome, 4-MU (Figure 2D). The benzopyrone structure of 4-MU responds to 350 nm excitation during the MUC assay and then is detected at 420 nm (Ziegler et al., 2000). Detection of 4-MU is the key step of the MUC assay and unfortunately, the phenolics and furans, added as inhibitors, shared similar molecular structure and absorbed energy around the same excitation wavelength of 350 nm (Piloto et al., 2006; Gómez-Alonso et al., 2007). Thus, in our tests, the interference caused by inhibitors with the excitation of 4-MU could have masked the detection of 4-MU. Figure 2D shows that sugar-rich fractions (F4, F5, and F6) did not affect the fluorescence of 4-MU (the same level as the amount of 4-MU produced in the recombinant enzyme control), whereas 1 mg/mL phenolics, 3 mg/mL furans and 4 mg/mL organic acid-rich fractions resulted in the loss of fluorescence of 4-MU by 9.8, 9.2, and 26.1% respectively. On the other hand, continued investigation using the cellulose-DNS assay also did not satisfactorily prove the inhibitory effects of the phenolics fraction (F1). Owing to its strong hydrophobic property, the phenolics fraction was maintained in butanol solvent. The butanol solvent (10%) was demonstrated to be a strong inhibitor of the cellulolytic enzymes, especially the cellobiohydrolases, during the cellulose-DNS assay (Figure 3E). For this reason, the butanol solvent could have caused the supposed inhibitory effects instead of the phenolics fraction. Further study is required to eliminate the impact of solvent-related inhibition.

Interestingly, investigation of F1, F2, and F3 using the cellulose-DNS assay showed that furans and organic acids did not induce any inhibition against the recombinant cellulolytic enzymes, and in the case of CBHI, they instead performed as enhancers. Furfural and formic acid were reported to be inhibitory to the *T. reesei* cellulases at concentrations above 5 mg/mL (Jönsson and Martín, 2016). The lack of inhibition observed in this work could be attributed to the fact that tested concentrations (1.5 mg/mL) of the CPC fractions F2 and F3 were low. It is also possible that the pinewood derivate could inhibit only at a higher proportion. In addition, since the HW hydrolysis is a benign pretreatment process, it did not produce enough furans or organic acids to inhibit the enzymes.

We also tested the efficiency of a commercial cellulase cocktail using the cellulose-DNS assay system in the presence of all CPC fractions. The efficiency of the commercial cellulase cocktail, which is a mixture of endo-cellulases and exo-cellulases among other enzymes, was significantly inhibited by the CPC fractions F5 and F6. Based on our earlier analysis we can conclude that it is more likely due to feedback inhibition of endo-glucanase, which in turn caused overall reduction in enzymatic efficiency. The effect of the phenolics fraction on the commercial cellulase cocktail remains unclear because of the existence of butanol, which also significantly inhibits the commercial cellulase cocktail (Figure 3E). Finally, furans (F2) were also moderately inhibitory to the commercial cellulase cocktail, which was unlike the individual recombinant enzymes, suggesting that the recombinant enzymes were more robust. Overall, oligomeric sugar-rich CPC fractions were inhibitory to individual cellulolytic enzymes (E1, CBHI, and CBHII) as well as to the commercial cellulase cocktail.

## Conclusion

Pinewood oligosaccharides were purified from the liquid fraction of hot water (HW) pretreatment using CPC. The results obtained in this work showed that along with other compounds, the liquid pre-hydrolyzate contained mannan, glucan, galactan, and xylan oligosaccharides, with mannan found in the highest amounts. These oligosaccharides presented different levels of impact on enzymatic efficiency of recombinant E1, CBHI, and CBHII, as well as the commercial cellulase cocktail. Higher DP oligosaccharides (DP > 5) are proved to be the most inhibitory compared to all tested CPC fractions. Inhibition conferred by other HW fractions, especially phenolics, was inconclusive because of their interference with cellulase assays. Whole liquid HW pre-hydrolyzate did not significantly inhibit either recombinant or commercial cellulolytic enzymes. Further studies focused on the mitigation of inhibition must be conducted, such that pinewood can be used to its full potential as a sugar source for sustainability-oriented biorefinery operations.

## Author contributions

HF doing main part of the experiment, data collection, interpreting the results, writing the first draft of the manuscript, and paper submission; GK, KR, AD, and DC participating some parts of the experiment and data collection, pinewood harvest, pinewood hot-compressed water pretreatment, CPC fractionation of the pinewood hydrolyzate, characterizing the pinewood hydrolyzate, and reviewing the manuscript; KH working on parts of the experiments; advisor for enzyme purification, participation in cellulolytic enzyme purification; EH funding recipient, mentor of this project, and reviewing the manuscript.

### Conflict of interest statement

The authors declare that the research was conducted in the absence of any commercial or financial relationships that could be construed as a potential conflict of interest.

## Abbreviations

AS: Ammonium sulfate
CBHI: 1,4-β-D-glucan-cellobiohydrolase I
CBHII: 1,4-β-D-glucan-cellobiohydrolase II
CPC: Centrifugal partition chromatography
DNS: Dinitrosalicylic acid
DP: Degree of polymerization
E1: Endo-1,4-β-D-glucanase
HMF: 5-hydroxymethylfurfural
HPLC: High performance liquid chromatography
HW: Hot water
LAPs: Laboratory analytical protocols
MU: 4-methylumbelliferone
MUC: 4-methylumbelliferyl-β-D-cellobioside
NREL: National Renewable Energy Laboratory
TFF: Tangential flow filtration
TSP: Total soluble protein.
